# Interactions Between Exercise Physiology and Metabolism

**DOI:** 10.3390/metabo16040229

**Published:** 2026-03-31

**Authors:** Lijing Gong, Chunyan Xu, Enming Zhang

**Affiliations:** 1 Key Laboratory of Exercise and Physical Fitness, Ministry of Education, Beijing Sport University, Beijing 100084, China; lijing.gong@bsu.edu.cn; 2 Key Laboratory for Performance Training & Recovery of General Administration of Sport, Beijing Sport University, Beijing 100084, China; 3 China Institute of Sport and Health Science, Beijing Sport University, Beijing 100084, China; 4 Beijing Higher School Engineering Research Center of Sport Nutrition, Beijing Sport University, Beijing 100084, China; xucy@bsu.edu.cn; 5 Department of Clinical Sciences in Malmo, Lund University Diabetes Centre, Lund University, 20213 Malmo, Sweden

Physical inactivity and excess nutritional intake have contributed to a global pandemic of metabolic and non-communicable diseases, resulting in unhealthy fat accumulation, metabolic dysregulation, and a high burden of chronic conditions such as type 2 diabetes, cardiovascular disease, and obesity [1]. Exercise, as a potent, non-pharmacological, and cost-effective intervention, orchestrates a complex network of physiological adaptations. It stimulates the release of a plethora of signaling molecules, including exerkines, which participate in inter-organ communication, regulate numerous key signaling pathways, and, in turn, enhance whole physical health, help prevent the development of chronic diseases, and actively help the body to recover [2]. The field of exercise metabolism seeks to capture these complex processes, and with the support of advanced omics technologies, it has revolutionized our ability to map the molecular landscape of exercise adaptations [3].

This Special Issue, entitled “Interactions between Exercise Physiology and Metabolism”, presents a compelling collection of eight original research articles that explore the multifaceted relationships between physical activity and metabolic health. The contributions cover a wide range of fields, ranging from large-scale epidemiological analyses in clinical populations to mechanistic studies in animal models and elite athletes. By utilizing state-of-the-art metabolomics, microbiomics, and epigenomics, these studies provide novel insights into how exercise modulates host metabolism, gut microbiota, and epigenes, as well as how these interactions influence health promotion and disease treatment.

**New Paradigms in Exercise Metabolism Research: From Traditional Indicators to Systems Biology**

Several studies in this Special Issue highlight the powerful potential of systems biology approaches in exercise metabolism research. Zhao et al. conducted a large-scale cohort study of 3373 urban elderly residents, employing statistical methods such as piecewise structural equation modeling (PiecewiseSEM) to systematically elucidate the mediating role of serum lipids in the relationship between physical activity (PA) and blood pressure (BP). Their findings revealed that the mediating effect of serum lipids was particularly significant under low-PA conditions. This discovery addresses the limitations of traditional studies that only focus on the direct association between PA and BP, offering a novel theoretical perspective for hypertension management (Contribution 1).

On the other hand, Fei et al. utilized untargeted plasma metabolomics to find that cardiorespiratory fitness (CRF) levels can mitigate the metabolic disturbances associated with metabolic syndrome (MetS) risk factors by modulating key metabolic pathways such as the TCA cycle and arginine biosynthesis. The eight common differential metabolites identified in this study (including branched-chain amino acids and 2-oxoglutarate) provide potential biomarkers for stratifying CRF levels and MetS severity (Contribution 2).

Collectively, these studies illustrate how macroscopic epidemiological analysis or microscopic metabolite profiling can yield new insight into the complex relationships between exercise and metabolism.

**Exercise Interventions and Metabolic Disease Management: From Mechanisms to Applications**

The role of exercise as a non-pharmacological intervention in metabolic disease management is strongly emphasized in this Special Issue. Varlet et al. conducted a large-scale calorimetry study of 6465 individuals, providing the first systematic characterization of the linear relationship between carbohydrate oxidation and exercise intensity, termed the “carbohydrate cost of the watt (CCW).” Their findings revealed that women, older individuals, and those with higher adiposity exhibited higher CCW values, suggesting a greater reliance on carbohydrates and relatively lower lipid oxidation capacity. This discovery provides a physiological basis for individualized exercise prescription in patients with obesity and metabolic diseases (Contribution 3).

Bathina et al. further investigated the mechanisms of lifestyle intervention (diet combined with exercise) from an epigenetic perspective. In severely obese men with hypogonadism, a 12-month lifestyle intervention significantly reduced global DNA methylation levels in peripheral blood mononuclear cells and downregulated the expression of key DNA methyltransferases (DNMT1, DNMT3A, DNMT3B) as well as adipogenic genes (PPARγ, CEBPα, FTO). This study provides novel evidence that lifestyle interventions can improve metabolic status through epigenetic reprogramming, offering a molecular explanation for the long-term benefits of exercise (Contribution 4).

**Training Modalities and Athletic Performance: Metabolic Adaptations and Individual Difference**

Studies involving athletic populations provide unique insights into the metabolic adaptation mechanisms of different training modalities. Kong et al. compared the effects of a threshold training model (72%:24%:4% intensity distribution) and a polarized training model (78%:8%:14%) on adolescent male rowers. Although both training modalities significantly improved 2 km rowing performance, the metabolic profiles they induced differed: the threshold group specifically enriched the pyruvate metabolism pathway, while the polarized group enriched the aminoacyl-tRNA biosynthesis pathway. This finding suggests that while both modalities are equally effective in enhancing aerobic endurance, they modulate through different molecular mechanisms, providing a metabolic rationale for scientific training (Contribution 5).

Fu et al. adopted a “sportomics” strategy to systematically monitor the multi-dimensional physiological status of collegiate sprinters during the pre-competition strengthening and tapering periods. Despite reduced training volume during tapering, athletes exhibited gut microbiome imbalance, elevated inflammatory factors (IL-6, IL-1β), and activation of immune-related metabolic pathways. These subtle physiological changes, not fully captured by traditional biochemical markers, were closely associated with athletic performance limitations, underscoring the unique value of multi-omics monitoring in elite sports (Contribution 6).

Li et al. further revealed the impact of sex differences on exercise-induced metabolic responses. Through serum metabolic profiling of freestyle wrestlers at different training stages, they observed that male athletes mainly exhibited increases in branched-chain amino acids and lactate during peak training, whereas female athletes showed decreased valine and increased pyruvate levels. This finding suggest that sex-specific metabolic responses should be fully considered in training monitoring (Contribution 7).

**The Exercise–Gut–Multi-Organ Axis: From Peripheral Metabolism to the Central Nervous System**

The gut microbiome has recently emerged as a critical mediator connecting exercise and systemic metabolism. The studies included in this Special Issue extend this perspective to the field of neural development. Meng et al. utilized a Shank3 knockout rat model of autism spectrum disorder (ASD) and provided the first evidence that early-life swimming intervention can reverse striatal metabolic abnormalities. Shank3-knockout resulted in significant reductions in neurotransmitters and neurodevelopment-related metabolites such as glutamate, glutamine, and taurine, whereas early swimming intervention restored these metabolite levels and improved glutamatergic and GABAergic synaptic pathways. This study offers a mechanics insight into the beneficial effects of exercise on ASD symptoms and supports the regulatory role of the exercise–gut–brain axis (Contribution 8).

Consistent with this perspective, Fu et al. also demonstrated that during the tapering period, athletes exhibited a decreased gut microbiome health index (GMHI), an increased microbial dysbiosis index (MDI), and alterations in inflammation- and immune-related metabolic pathways. These findings further support the gut microbiome as a sensitive indicator of physiological responses to exercise (Contribution 6).

**Conclusions and Future Perspectives**

The studies presented in this Special Issue collectively provide a multifaceted and comprehensive view of the interactions between exercise and metabolism. Encompassing large-scale population cohorts, clinical metabolic diseases, elite athletes’ performance, and neurodevelopmental disorders, and integrating approaches from traditional biochemical assessments to advanced multi-omics analyses, these contributions significantly deepen our understanding of exercise metabolism mechanisms.

Future research should prioritize the integration of multi-dimensional data, including metabolomics, metagenomics, and epigenomics, combined with advanced analytical techniques such as artificial intelligence, to develop predictive models for individualized exercise prescriptions. Such efforts will facilitate the transformation of exercise interventions from a “one-size-fits-all” approach toward precision and personalization. Moreover, enhancing the translation of basic research findings into clinical practice and validating the utility of exercise-related metabolic biomarkers in disease prevention and management will be essential for addressing the growing global challenge of metabolic diseases.

## Data Availability

No new data were created or analyzed in this study. Data sharing is not applicable to this article.
